# Protocol for measuring context-dependent cost-benefit decision-making in humans using a web application

**DOI:** 10.1016/j.xpro.2025.104077

**Published:** 2025-09-12

**Authors:** Lara I. Rakocevic, Raquel Ibáñez Alcalá, Ki A. Goosens, Alexander Friedman

**Affiliations:** 1 Computational Science Program, University of Texas at El Paso, El Paso, TX, USA; 2 Department of Biological Sciences, University of Texas at El Paso, El Paso, TX, USA; 3 Department of Psychiatry, Center for Translational Medicine and Pharmacology, Friedman Brain Institute, Icahn School of Medicine at Mount Sinai, New York, NY, USA

**Keywords:** Neuroscience, Cognitive Neuroscience, Behavior, Computer sciences

## Abstract

We present a protocol for measuring naturalistic and normalized decision-making in humans across four contexts (approach-avoid, moral, social, and probabilistic) using a web application. We describe steps for session setup, eye tracker calibration, and heart rate monitoring. In each session, a participant encounters a story, rates rewards and costs relevant within that context, and then evaluates various cost-reward pairings in context.

For complete details on the use and execution of this protocol, please refer to Rakocevic et al.^1^

## Highlights

• Procedures for deploying a context-dependent decision-making web application
• Steps for extension to new scenarios and types of decision-making
• Instructions for tracking eye movement and heart rate for each choice
• Database setup instructions for automatic uploading of participant responses

## Abstract (editor highlights)

Publisher’s note: Undertaking any experimental protocol requires adherence to local institutional guidelines for laboratory safety and ethics.

## Graphical abstract

**Figure:**
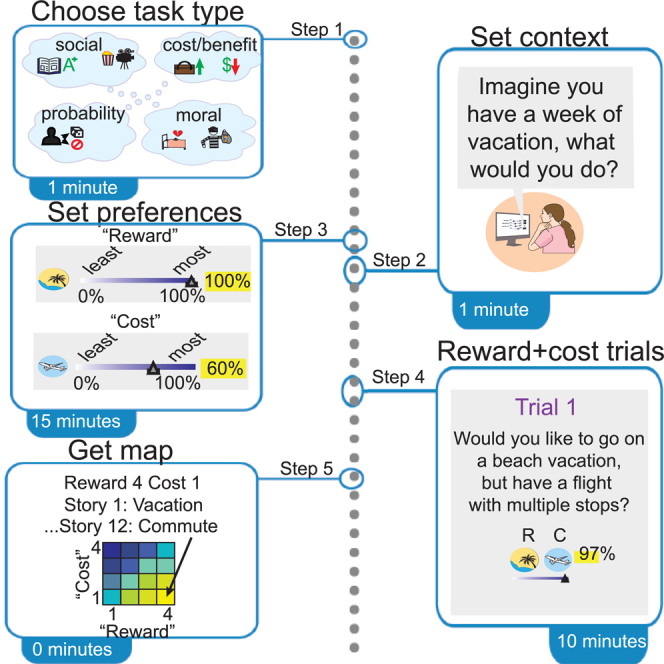

## Before you begin

We present a cost-benefit decision-making task that examines individual decision-making using stories as a vehicle for delivering costs and rewards and is designed for psychometric analysis. We developed stories spanning a wide range of contexts approach-avoid, social, moral, and probabilistic in order to examine and compare decision-making under unique circumstances. This paper is a protocol for a new method described in depth in Rakocevic et al.^1^

Examining human decision-making has been a topic of interest that has been executed in various forms. Some of these tasks include those that explore risky decision-making^2^ like the Iowa gambling task^3^^,^^4^ or balloon analog risk task.^5^^,^^6^ Other tasks examine aspects of social decision-making^7^^,^^8^^,^^9^ or neuroeconomic decision-making.^10^^,^^11^ Many of these experiments are excellent for examining separate aspects of decision-making, however being able to compare directly across contexts within a singular environment would prove invaluable for future research. Reading stories and imagining situations is a natural process for humans and this naturalistic task leverages this ability to examine a wider range of contexts. In particular, leveraging stories to examine different kinds of decision-making allows for additional flexibility and fast iterations. Instead of developing additional tools to dispense rewards and costs, one must only write a story. If additional contexts are needed, they may be easily added.

In addition, this task also allows us to measure psychometric functions, examining multiple reward levels and multiple cost levels. Finally, we normalize the costs and rewards to each individual subject, so this task only presents costs/rewards directly relevant to the subject which is a unique to this paradigm. In addition, we wanted to develop an application that could be easily deployed on any computer or phone to measure decision-making on a daily basis. This is especially critical for the use in psychological contexts, and measuring how an individual’s decision-making may change from day to day. This application gives us the ability to measure psychological changes in an ongoing way due to its flexible implementation.

### Protocol overview

**Figure 1:**
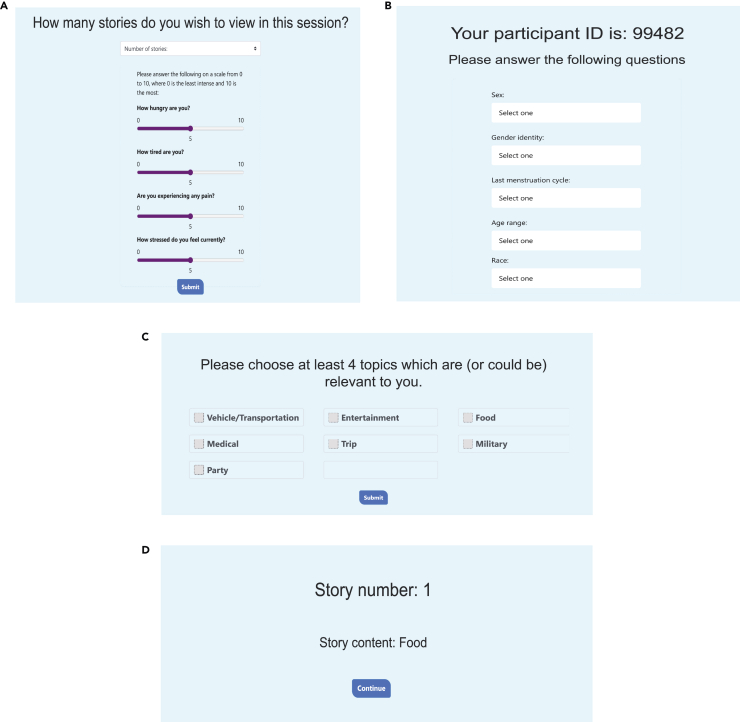
Visuals of the application: Metadata and story selection (A) At the beginning of the session, the subject or moderator inputs the number of stories that the subject will complete in the current sessions. The subject also will also answer questions regarding their physical state, including their levels of hunger, tiredness, pain, and stress. (B) The greeting screen for first-time participants. Each participant is given a randomized ID number that the participant’s data will be associated with in the database. We also collect metadata at this time. (C) Participants select which story topics are relevant to them so that the application only presents stories that the subject can respond to accurately and with care. (D) Once story types have been selected, a random story with a relevant topic will be presented.

**Figure 2:**
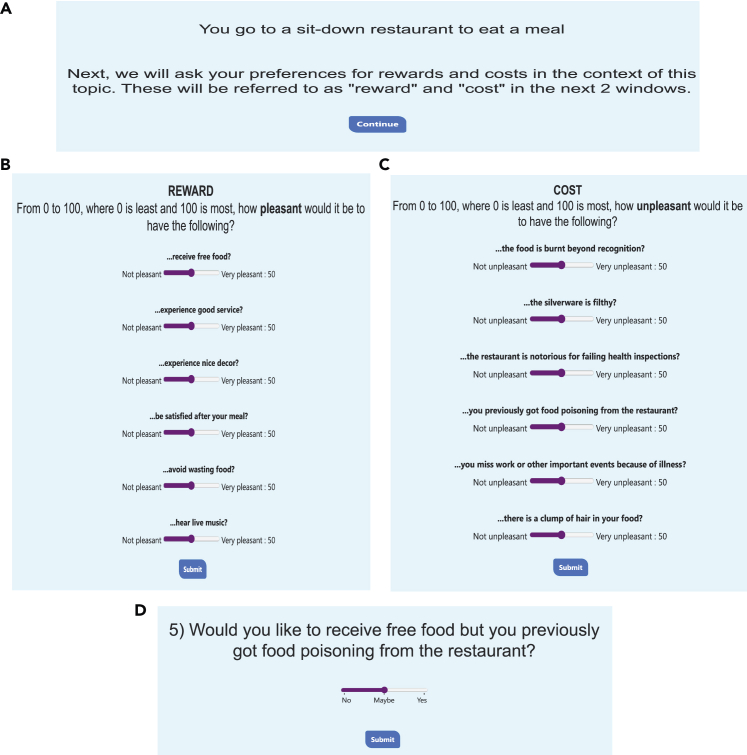
Visuals of the application: Preferences and trials (A) The story context is presented. In this case, the story topic was “food” and the context presented gives the subject a situation surrounding eating food at a movie theater. This allows the subject to evaluate the rewards and costs presented in the following panels within the context of the story. (B) Six options for potential rewards are presented to the subject relating to the story context. The subject must evaluate each from 0 to 100, based on how pleasant the reward would be. No two options may have the same value. (C) Similarly, six options for potential costs are presented to the subject relating to the story context. The subject must evaluate each from 0 to 100, based on how unpleasant the cost would be. (D) The application chooses four of the six rewards and four of the six costs, choosing rewards and costs that have equidistant spacing in value and span the entirety of the point space. Each reward and cost is assigned a level from 1 to 4. Then, each reward is paired with each cost and presented in a random order. Participants respond with how likely they are to accept the offer on a sliding scale from 0 to 100, from “never accept” to “always accept”.

**Figure 3:**
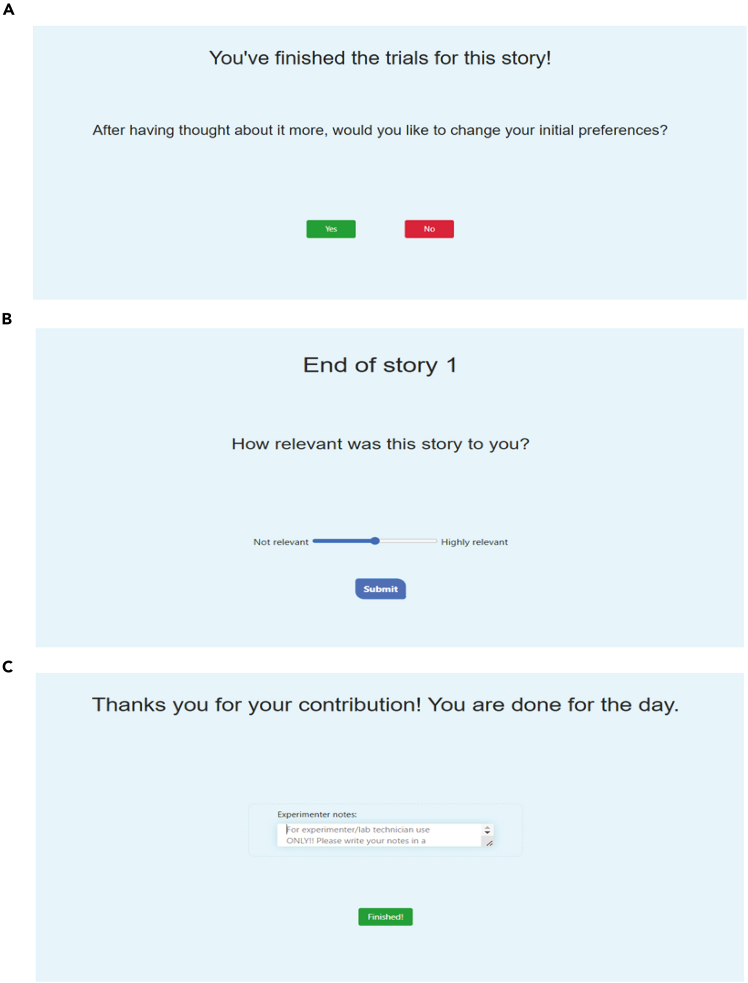
Visuals of the application: End of story and session (A) Once all 16 trial questions have been answered, the story concludes. At this point, subjects have the choice to re-evaluate their initial reward and cost preferences if they found that they misjudged their preferences after completing the trials. If so, they are taken back to the same preferences page as before and both the old and new preferences are noted in the database. (B) Subjects are asked to rate how relevant the story was to them on a scale of 0 to 100. (C) Before moving on to the next subject, the moderator of the experiment has the ability to make notes about the experiment, which will be uploaded to the database with the subject’s data.

Before the session begins, demographic and state information is collected about the subject, including hunger and tiredness (Figure 1A). Subjects then choose what types of stories they would be interested in answer (Figure 1B). In each session, subjects are presented with a story context that they will answer questions about (Figures 1C and 2A). First, the subject is presented with a list of 6 reward (Figure 2B) and 6 cost levels (Figure 2C) that are related to the story context and must evaluate these preferences, assigning each potential reward and cost with a rating from 0 to 100 depending on how preferable or unpreferable the option is. From there, 4 rewards and 4 costs are selected algorithmically in such a way that the ratings given by the participant are as equidistant as possible. These 4 rewards and 4 costs are all paired to create 16 offers, which are then presented to the subject in a random order (Figure 2D). The subject then responds with how likely they are to accept the offer within the given context, from 0 to 100. If a subject would like to adjust their preferences after completing a story, they may do so (Figure 3A), otherwise the story ends and the subject can answer how relevant the story was to them (Figure 3B). As responses are provided, the results of the experiment are automatically written to a PostgreSQL database where each trial with a distinct reward and cost pairing within a session will be an entry in the database. The moderator of the experiment can leave any notes as well before the session closes (Figure 3C), and this will also be uploaded to the database.

### Story design

We use stories as a tool to deliver levels of reward and cost to consider for making a decision. The stories we have written and provide with the application were designed for a student population, but can be modified or changed for another audience. In general, we chose these specific stories because they represent every-day kinds of decision-making that students may encounter and would capture naturalistic decision-making for this population.

If the stories are not relevant and the costs and rewards are meaningless to an individual, then the responses will be skewed. On the flip side, if the questions are too sensitive and the decisions too controversial, it may be difficult for the participants to answer. Therefore, it is important to make sure that the stories used are relevant and clear to the population you wish to examine. We recommend selecting stories that your subject population would encounter on a regular basis, so that they can easily imagine the scenarios and can respond with a full understanding of the situation being presented.

In addition, it is important to make sure that stories are properly categorized across task types. For example, social situations often have a probabilistic element to them (i.e. “My friend *might be* mad at me”). In order to properly differentiate between task contexts, it is critical that stories are written precisely and in such a way that they do not overlap multiple contexts.

Using a power analysis to measure the difference in reward approach across levels or cost avoidance across levels, we found that to find a significant difference between approach rates in the reward level 1 and reward level 4 you need 8 subjects and to find a significant difference between cost level 1 and cost level 4 you need 20 subjects. Therefore, we recommend collecting at least this many stories per subject to yield a successful decision-making map with clear differences in approach rates across varying levels of reward and cost.

### Data collection

In order to collect the requisite number of stories, data collection may need to be split across several days. In this case, it is important to consider the baseline state of the subject changing across days due to physiological factors like hunger, tiredness, pain, stress, etc. All of these factors are known to impact decision-making and therefore may cause differentiated decision-making. In our application, we collect metadata like these physiological factors per session, so it will be included in the database, but may affect expected outcomes.

### Institutional permissions

Human data collection for this project received approval for all protocols from the University of Texas at El Paso IRB on June 19, 2024 (FWA No. 00001224).

### Database setup

**Timing: 30 min**

1. This application is defined to run on Windows (Version 10 or higher).

***Note:*** To run on Mac or Linux, additional adjustments may need to be made.

2. Ensure that you have PostgreSQL (Version 14 or higher), MATLAB (Version 2023b or higher), and Python (Version 3.10.5) properly installed. When installing, accept all default values in the installation process.
3. To Set all the variable paths set on your machine. To set the path for PostgreSQL,
  a. Press **Windows**, type **“Environment Variables”**, and open: **Edit the system environment variables**.
  b. In the **System Properties** window, click **Environment Variables.**
  c. Under **System variables**, find and select the **Path** variable, then click **Edit.**
  d. Click **New**, and paste C:\Program Files\PostgreSQL\14\bin (or enter the user’s path to PostgreSQL.
4. Download a copy of the database backup, located at the following link: Harvard Database: https://doi.org/10.7910/DVN/OZARPL Unzip the file into a file on your local machine.
  a. The .zip contains two files:
    i. a .zip named ‘Database Backup As Of July 4th 2024.zip’ that itself contains the .tar.
    ii. a folder named ‘physiological data’.
  b. Unzip ‘Database Backup As Of July 4th 2024.zip’. This contains a .tar file.
5. Open the windows command line (ensure that you open it as an administrator).
6. Connect to the PostgreSQL server, using the following command:

psql -U postgres

7. Create a new empty database, using the following command:

create database live_database;

8. To check the database has been successfully created, use the command “\dt”.
9. To exit psql, using the command “\q”.
10. Restore the database from the backup .tar file, modified to suit local file paths, using the following command, replacing the file path in quotes below to the actual path where the tar file is located locally:

   pg_restore -U postgres -d live_database

« C:\Users\username\folder\dataverse_files\Database Backup As Of July 4th 2024\backupFile1.tar »

***Note:*** Runtime may extend past 10 min depending on RAM and CPU.

11. Connect to the live_database using:

psql -U postgres -d live_database

### Application setup

**Timing: 15 min**

12. Ensure that you have Git properly installed on your machine.

***Note:*** If unfamiliar with Git, use the following guide to get started: https://docs.github.com/en/get-started/start-your-journey/hello-world.

13. Git clone the application from our repository using the following code:

git clone https://github.com/rjibanezalcala/HUMANS.git

14. Adjust application settings:
  a. Navigate to the directory where you extracted the decision-making app files to.
  b. Navigate to the ‘bin’ folder and open ‘settings.ini’ with your text editor of choice (Notepad, Notepad++, Atom, etc…).
  c. Enter the PostgreSQL database credentials.
    ***Note:*** This will look different depending on how the database was set up. For instance, if the user accepted default settings when installing PostgreSQL, change the values in the lines 4 to 8 to:
    Host=localhost
    database=live_database
    port=5432
    user=postgres
    password=YourRealPasswordHere
  d. Change the settings that will affect how the app behaves.
    ***Note:*** Settings related to heart rate and eye-tracking will be detailed below, and other settings are described in the supplemental materials.
15. Once the necessary changes have been made, save the file and exit the text editor.

### Heart rate monitor setup

**Timing: 30 min**

***Note:*** Collecting heart-rate data is an optional part of running this application and connecting a device is not necessary for running the application.

***Note:*** We recommend using the OpenANT Heart Rate Monitor. If you wish to use a different device, then you must update the file “heartrate_lib.py” (https://github.com/rjibanezalcala/HUMANS/blob/main/heartrate_lib.py) to be compatible with the hardware you wish to use.

16. To collect heart rate data using a desktop or laptop computer and a chest strap heart rate monitor (HRM, for example item 12 in the key resources table), install the ANT+ USB driver for Windows (item 2). A guide is listed under item 3.
17. Connect the ANT+ USB antenna (item 15) to your PC and power up your HRM.
18. If you are using an external program to collect heart rate data, follow the steps below. If you are instead using the HRM thread built-in to the app, skip to step 4.
  a. Make sure your program of choice is compatible with ANT+ devices; we have listed recommended software under item 13 in the key resources table.
  b. After installing your program, be sure to set up your device following the software’s requirements. The following are setup steps using this software.
  c. Open Pulse Monitor and click the ‘Devices’ button.
  d. Enter the heart rate monitor’s serial number and name in the corresponding dialogue boxes,
  e. Click the ‘Scan for devices’ button and wait for your device to appear. Once your device appears, click the ‘Add’ button.
  f. Close the dialogue box when you’re done.
  g. Navigate to the directory where you extracted the decision-making app files to. In the ‘bin’ folder, open ‘settings.ini’ with your text editor of choice (Notepad, Notepad++, Atom, etc…).
  h. Under the ‘hr_tracker’ section, change the following items:
    i. use_external_app=1.
    ii. copy your heart rate monitoring app’s install directory path to the external_app_install_path parameter (for example ‘D:\Program Files\PulseMonitor’).
    iii. use_hrtracker=1.

***Note:*** This tells the app to open the linked software as a subprocess. Keep in mind that this program will run asynchronously alongside the app, thus the data produced by this program will not be directly uploaded to your database. The produced data must be exported from the HRM software, saved, preprocessed, and then uploaded to the PostgreSQL database (see “database setup”)

19. If you are using the built-in HRM, follow the steps below:
  a. Navigate to the directory where you extracted the decision-making app files to.
  b. In the ‘bin’ folder, open ‘settings.ini’ with your text editor of choice (Notepad, Notepad++, Atom, etc…).
  c. Under the ‘hr_tracker’ section, change the following items:
    i. use_external_app=0.
    ii. use_hrtracker=1.
    iii. hrtracker_index=0 (set to 0 if you only have one heart rate monitor connected, otherwise specify your device’s index)
    iv. emulate_device=0.
    v. run_thread_as_daemon=1 (optional).
    vi. verbose=0 (optional, can be set to 1 if you want the heart rate monitor output displayed on the command line)
    vii. test_on_startup=1 (optional).
  d. Save the file and exit the text editor.

***Note:*** These settings will ensure that the HRM data are directly collected by the app and uploaded to the database. No additional steps are required.

### Eye tracker setup

**Timing: 30 min**

***Note:*** Collecting eye-tracking data is an optional part of running this application and connecting a device is not necessary for running the application. If you wish to collect eye-tracking data, we provide details for the hardware and software setup.

***Note:*** The following setup is for the Tobii Eye Tracker. If you wish to use a different software device, then you must update the file “eyetracker_lib.py” (https://github.com/rjibanezalcala/HUMANS/blob/main/eyetracker_lib.py) to be compatible with the hardware you wish to use. In addition, setup steps may vary. Refer to the user guide of the hardware device for device-specific details.

20. Download and install items 4, 5, and 7 from the key resources table.
21. Mount your tracking device (item 16) following the manufacturer’s guidelines (item 6).
22. Connect the device to any available USB port, preferably one directly on the computer tower and not through a USB hub, as this could cause issues with data throughput.
23. Test the eye tracker by running the eye tracker manager (item 7). Your eye tracking device should show up in the list of available devices at the top.
24. Click your device and the user interface (UI) will expand. Toggle both ‘gaze visualisation’ and ‘position guide’, then look around and see if the gaze visualizer moves to where you are looking. If it doesn’t, click the ‘Calibrate’ button and follow the calibration steps shown on screen.
25. Navigate to the directory where you extracted the decision-making app files to.
26. In the ‘bin’ folder, open ‘settings.ini’ with your text editor of choice (Notepad, Notepad++, Atom, etc….
27. Make the following changes under the ‘eye_tracker’ section:
  a. manager_install_path=C:\Users\{YOUR_WINDOWS_USERNAME}\AppData\Local\Programs\TobiiProEyeTrackerManager\TobiiProEyeTrackerManager.exe.
    ***Note:*** This may differ depending on where you installed the Eye Tracker Manager program.
  b. subscriptions=[‘gaze’,’position’].
    ***Note:*** “Openness” is also valid; however, it may not be supported by your device.
  c. eyetracker_index=0.
    ***Note:*** Set this to 0 if you have only one eye tracker connected, otherwise specify your device’s index.
  d. use_eyetracker=1.
28. Save the file and exit the text editor.

## Key resources table

**Table:** 

REAGENT or RESOURCE	SOURCE	IDENTIFIER
**Software and algorithms**		

HUMANS App Github Repository ∗		https://github.com/rjibanezalcala/HUMANS
ANT+ USB Driver (Windows)	ANT+	https://www.thisisant.com/developer/resources/downloads/, accessed June 2025
ANT+ USB Driver installation guide (Windows)	ANT+	https://support.wahoofitness.com/hc/en-us/articles/360021559679-Installing-ANT-drivers, accessed June 2025
Tobii Pro SDK ∗∗∗	Tobii	https://www.tobii.com/products/software/applications-and-developer-kits/tobii-pro-sdk, accessed June 2025
Tobii Pro Spark Runtime Driver ∗∗∗	Tobii	https://connect.tobii.com/s/spark-downloads?language=en_US&tabset-96e49=3, accessed June 2025
Tobii Pro Spark User Guide ∗∗∗	Tobii	https://go.tobii.com/tobii-pro-spark-user-manual, accessed June 2025
Tobii Eye Tracker Manager ∗∗∗	Tobii	https://www.tobii.com/products/software/applications-and-developer-kits/tobii-pro-eye-tracker-manager, accessed June 2025
Python 3.10.5		https://www.python.org/downloads/
Tobii-research 1.11.0		https://pypi.org/project/tobii-research/
openant 1.2.1		https://pypi.org/project/openant/
Flask 2.2.3		https://pypi.org/project/Flask/
PostgreSQL 14.1		https://www.postgresql.org/download/
Pulse Monitor	https://www.pulsemonitor.net/, accessed June 2025	

**Other**		

ANT+ Heart Rate monitor (HRM) ∗∗	ANT+	https://www.wahoofitness.com/devices/heart-rate-monitors/tickr-buy, accessed June 2025
ANT+ USB Antenna	ANT+	https://www.wahoofitness.com/devices/indoor-cycling/parts-components/usb-ant-kit-buy, accessed June 2025
Eye tracker	Tobii	https://www.tobii.com/products/eye-trackers/screen-based/tobii-pro-spark, accessed June 2025
Alcohol wipes		
Keyboard and mouse

***Note:*** ∗This guide pertains to only the code contained in the main branch of our GitHub repository. The code contained in any other branch is not documented in this guide. Please ensure you are downloading code from the main branch.

***Note:*** ∗∗Another ANT+ HRM can be used, but none were ever tested.

***Note:*** ∗∗∗These items may vary depending on what eye tracking system you are using. In this guide, we assume the Tobii Pro Spark eye tracker, and all links in this guide were made with that in mind. If your eye tracking system differs from ours, consult the manufacturer’s instructions for setup and installation.

## Step-by-step method details

In this section, users prepare the experimental set-up and decide how many stories participants will be answering, what kinds of stories, and if participants will be using a heart-rate monitor or eye-tracking device.

### Session setup

**Timing: 5 min**

1. In “settings.ini”:
  a. Adjust line 76-77 to “1” if tracking eye movement or “0” if not.
  b. Adjust line 88-89 to “1” if tracking heart-rate or “0” if not.
2. Open the folder “decision-making-app”. In this folder, double click on the batch script named **‘startup.bat’** in the DM app folder.

***Note:*** This will open a command line window, which must remain open as long as the app is running. This window will display information essential for troubleshooting.

3. Before beginning a session, prepare the subject for the session by doing the following:
  a. Ask the participant for any relevant information you may need in private, make sure to store this information in a secure location. This may include:
    i. Consent forms.
    ii. Name to be associated with ID numbers for bookkeeping purposes.
    iii. Eyesight conditions that may require corrective lenses.
    iv. Use of pace-makers or health conditions that could complicate collection of heart rate data.
    v. Whether they choose to not wear a heart rate monitor.
4. If this is the participant’s first session, give them a run-down of what they can expect from the recording session, including details about eye-tracking.

***Note:*** The eye tracker is not programmed to capture any images during the trial. Only gaze (where the subject is looking on the screen), pupil dilation, and user position in front of the tracker are collected and recorded.

***Note:*** Make sure that they know that they may choose not to disclose certain demographic information that the app asks for, and that their data will only ever be identified by a randomly generated ID number and not by their name.

5. Allow the participant to answer the questions in the first screen of the app, including information about hunger, tiredness, and other information about their current physical state.

### Eye tracker calibration

**Timing: 5 min**

In this section, users that are using a eye-tracking device calibrate the eye-tracker for the participant.

***Note:*** If using a different eye-tracking device, please refer to the manufacturer’s instructions for setup and installation. The following steps are relevant only for the Tobii Pro Spark Eye Tracker.

6. Upon continuing to the second screen, two programs will run. Bring only the **Tobii Eye Tracker Manager** window to focus. Click on the button that says ‘Pro Spark’ on the top of this window.
  a. If Tobii Eye Tracker Manager does not open, stop and check that the eye tracker is connected to the computer and that the ‘use_eyetracker’ option is set to 1 in ‘\bin\settings.ini’.
  b. The app will ask you (through the command window) if you wish to continue without the eye tracker.
    i. Type ‘Y’ and press Enter on your keyboard to continue with the eye tracker disabled (not recommended); or type ‘N’ to exit the app and troubleshoot.
7. Click the ‘Calibrate’ button. Instructions will show up on-screen and calibration will begin.

***Note:*** Every subject should go through the calibration process at least once before starting the trials. Remember that eyeglasses or contact lenses must be worn during this process for calibration to be accurate!

8. After calibration is done, close the **Tobii Eye Tracker Manager** window and the app will continue.

### Are you a new participant?

**Timing: 1 min**

In this section, new participants get their randomly generated ID number that they must share with the moderator of the experiment.

9. Allow the participant to click through the next screen (‘Are you a new participant?’).
  a. If the subject is a new participant, take note of their ID number and ask them to do the same. They will need this number to ‘sign-in’ for subsequent sessions.
  b. If the subject is not a new participant, either the moderator of the experiment or the subject may enter the subject’s ID number in the text box.
10. If the subject is wearing the HRM and/or ‘use_hrtracker’ in ‘\bin\settings.ini’ is set to 1, continue to step 11, otherwise skip to step 12.

### Heart rate monitoring

**Timing: 5 min**

In this section, users that are using a heart-rate monitor set it up for use by a new participant.

***Note:*** If you are using external HRM software (f.e. Pulse Monitor) to collect heart rate data, follow the following steps, otherwise skip to step 12.

11. If the subject is a new participant, register them into the **Pulse Monitor** software by clicking the ‘Participants’ button.
  a. Click on ‘Add Participant’ and allow the subject to input their information; ask them to enter their generated ID number (from the web browser) under ‘name’ and ‘pseudo’. Click ‘Save’ when done.
  b. Click on ‘Start New Training’. Add the participant’s ID number to the list of participants in the ‘workout’ if it is not already there.
  c. Choose ‘Wahoo’ (or your HRM device’s name) from the ‘Device’ dropdown menu. Click ‘Add’ and ‘Go to practice’.
  d. Start the ‘workout’, click the small gray button on the bottom right of the screen, and set this window aside.

### Session start

**Timing: 10 min**

In this section, the session is run and participant data is collected.

The session will start as soon as the first ‘Context’ screen is shown. After this, the subject’s preferences will be collected, and a list of questions will be generated for the subject to answer.

***Note:*** Eye tracking data will only be collected while the subject is on the question screens.

### Session end

**Timing: 1 min**

In this section, the session is completed and final notes are taken.

12. The ‘You are done for today!’ screen indicates the end of the session. Ask the subject to step away from the computer at this time.
13. Write down any notes about the session you wish to let the data analysts know. Such notes may include but are not limited to:
  a. Interruptions due to environmental factors.
  b. Any comments that the participant may have had.
  c. Catastrophic app failures.
  d. Having to split the session into two.
14. Click on the green button that reads ‘Finished!’.

### Cleanup and heart rate data export

**Timing: 3 min**

In this section, users do final housekeeping after the end of the experiment and clean up any devices that were used.

15. Ask the subject to remove the heart rate monitor.
16. If you are using external HRM software to collect heart rate data, do the following:
  a. Stop the ‘workout’ on the Pulse Monitor software.
  b. Export the subject’s data by right clicking on their session summary and clicking ‘Save full HR data’. This will export a .csv file with timestamped heart rate data.
  c. Save this file under the directory named after the participant’s ID number in ‘\data\’ with the following format: *‘{participant ID}_{mm}-{dd}-{yyyy}_{hh}-{mm}-{ss}.csv’*
  d. To upload the HRM data to the database, use the database injector script contained in the ‘/helper_scripts/Database_Injector’ folder (https://github.com/rjibanezalcala/HUMANS/tree/main/helper_scripts/Database_Injector). See the README file for instructions on how to use this script.
17. Wipe down the heart rate monitor with alcohol wipes for the next participant to wear.
18. If ‘enable_consecutive_users’ is set to 1 in ‘\bin\settings.ini’, the app will be ready to take the next subject. Otherwise, close the command window, run **‘startup.bat’**, and refresh the app in the browser window.

## Expected outcomes

Across stories, rewards (and costs) assigned to the same level should have comparable ratings, which allows us to compare trials with the same rewards and cost combinations across different stories (i.e. trials with reward level 1 and cost level 1 are comparable across sessions). This step of normalizing rewards and costs across subjects and stories allows us to compare subjects’ decision-making strategies more effectively across individuals and contexts. In addition, having multiple levels of reward and cost allows fit to a psychometric function on the subject responses, optimizing for psychometric analysis.

**Figure 4:**
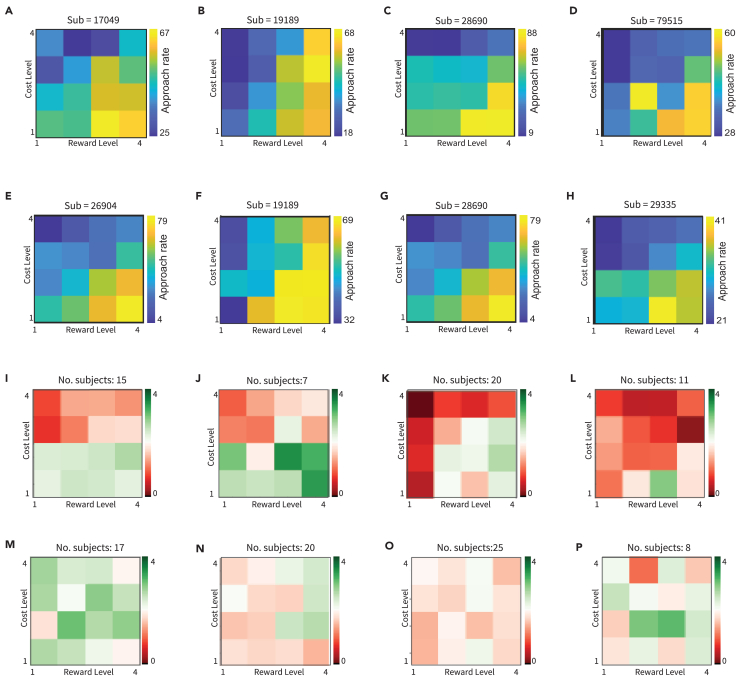
Decision-making grids and normalization (A–H) Decision-making maps per subject and context. All stories that a subject completed within a specific context (approach-avoid, social, moral, or probabilistic) are aggregated and averaged into one decision-making map showing the subject’s approach rates across varying levels of reward and cost. (I–L) Examples of the decision-making maps for individual stories, created by aggregating responses from all participants who completed that story. These examples represent successful maps, with higher approach in the bottom right corner (high reward-low cost) and higher avoid in the upper left corner (high cost-low reward) (M–P). Examples of decision-making maps for individual stories, created in the same way as in examples (I–L). These examples represent unsuccessfully calibrated stories.

**Figure 5:**
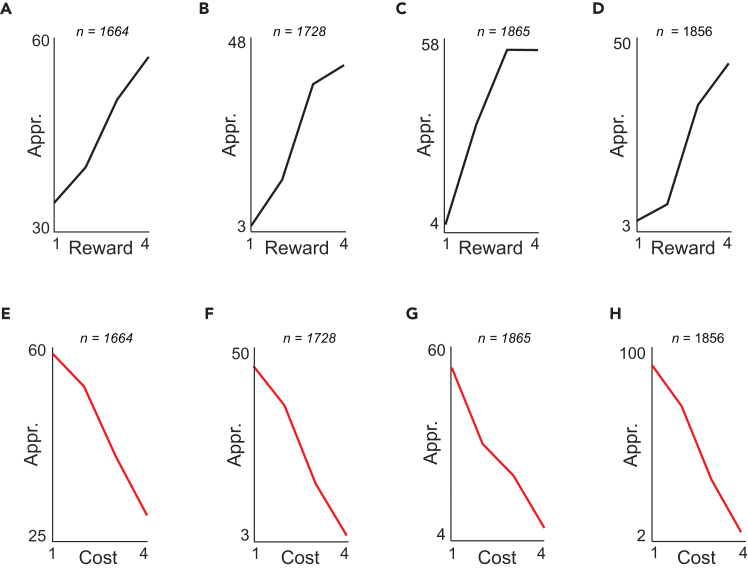
Task validation: Approach rates across cost/reward levels (A–D) Average approach rates across all subjects and stories increase as reward levels increase across all approach-avoid sessions (A), social sessions (B), probabilistic sessions (C), and moral sessions (D). (E–H) Average approach rate increase as reward levels increase across all approach-avoid sessions (E), social sessions (F), probabilistic sessions (G), and moral sessions (H).

In order to visualize the combination of rewards and costs in a more integrated way, we use “decision-making maps” (Figures 4A–4H). In these maps, we expect that the lower right corner will be more yellow (“approach-heavy”) and the upper left corner will be more blue (“avoid-heavy”) though the actual gradation of colors will vary with individual responses. We define a “decision-making boundary” by fitting a function to the approach rates, which separates offers from “mostly approach” and “mostly avoid”. Each story takes about 10 minutes for the subject to complete. We recommend including 4 stories in a session, otherwise subjects become tired, therefore the subject should spend around 40 minutes completing the task within each session. When creating new stories, it is also useful to create decision-making maps for each story to visualize how subjects are responding to the story, both successfully (Figures 4I–4L) and unsuccessfully (Figures 4M–4P). Code for creating decision-making maps and performing other analyses using this data can be found in the corresponding methods paper.^1^ We expect in general that as the reward level increases, approach rates will increase, and that as cost level increases (Figures 5A–5D), approach rates will decrease (Figures 5E and 5F).

### Tracking physiological features

**Figure 6:**
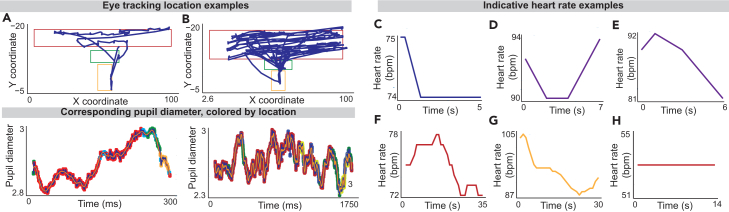
Optional eye-tracking and heart-rate monitoring outcomes (A and B) Eye-tracking data for a single trial shows how a participant’s gaze moved over the course of the trial, as well as the diameter of their pupils throughout the task. The boxes on the positional data indicate the location of the question text (red), the approach-rate slider (green), and the submit button (yellow). These positions are also indicated on the pupil diameter data by the corresponding colors. Two distinct trials are shown with examples of the extracted features, including: average pupil diameter, local maxima in pupil diameter, local minima in pupil diameter, the reading period, and the decision-making period. We use the Tobii Pro Spark eye tracker to track eye movement. (C–H) Heart-rate (HR) data for a single trial shows how a subject’s beats per minute (bpm) changes over the course of the trial. Individual examples are selected to show various types of examples including sudden dips in heart-rate (C and F), increases across the duration of a trial (D), decreases across the duration of a trial (E and G), and steady heart-rate across a trial (H). We use the OpenANT pulse monitor to track heart rate.

The expected outcome for physiological tracking is that eye movement and heart rate data are uploaded to the database in tandem with each trial. Heart rate data is uploaded as a list of beats per minute (BPM) values across the length of the trial. Eye-movement data is uploaded as a JSON containing various features, including pupil diameter through the trial and coordinates of where the left and right eye were looking through the trial. Examples of the pupil diameters and coordinate location of gaze on the screen throughout the trial (Figures 6A and 6B) and heart rates (Figures 6C–6H) can be seen in Figure 6.

## Limitations

The primary limitations involved in using this application relate to our reliance on stories. We use imagination as a vehicle for delivering costs and rewards. Therefore, it is critical that the participants are able to imagine themselves in the situations described and have familiarity with the contexts, rewards, and costs being presented to them. If the stories are not suited to the population, then the responses will be rendered meaningless. It is crucial to write or use stories that the subjects understand. This includes making sure that the subjects are fluent in the language that the story is written in as well.

Before collecting data using new stories, it is important to pre-test the stories. It is essential to balance the rewards and costs presented: if the costs out-weigh the rewards substantially, or vice versa, the data will be skewed and it will become difficult to separate out the participant’s decision-making tendencies from the story’s skew. One way to check this would be to look at the outcome of the decision-making map across individuals completing the same story. An ideal outcome would be a balanced boundary, with avoidance increasing with increasing cost and approach increasing with increasing reward (Figures 4I–4L). If the story is not calibrated well, the decision-making map will show no distinct patterns (Figures 4M–4P). We had 10 members of our team complete each story ahead of time and then aggregated the responses into a single decision-making map to check that these expectations were met.

In addition, when writing new stories, it is important to check that every reward and every cost can be paired with each other. If a reward and cost are chosen, but do not make sense when put together, then the offer is rendered meaningless. Similarly, when writing questions, it is important to offer the reward and cost exactly as first presented to and rated by the subject. If the question presents a slightly different reward or cost, then the initial rating given by the subject may not apply.

Finally, it is important to categorize stories properly. As mentioned previously, social stories can easily become probabilistic if imprecise language is applied (i.e. “I *might* miss my friend’s party.”). When adding and writing new stories, it is important to make sure that it is only approach-avoid, moral, social, or probabilistic, especially if studying effects across task contexts.

## Troubleshooting

### Problem 1

Receiving a “ValueError”, “Empty preference options”, or “IndexError”. This may occur during the session, between session set-up steps 11 and 12.

### Potential solution

These errors happen because ‘pref_reward.txt’ or ‘pref_cost.txt’ were formatted incorrectly or otherwise had a typo. They can be fixed by going directly to these files and editing any mistakes, then saving the file and refreshing the page.

### Problem 2

Occasionally, the code raises an exception that cannot be recovered from by refreshing the page. This is usually due to either external hardware preventing code from running, or critical data was pre-loaded into memory and cannot be re-created using new information. This may occur during the session, between session set-up steps 11 and 12.

### Potential solution

• You may ask the subject to skip a question altogether and continue through the rest of the trials. However, the subject may encounter the same error again if there was more than one question that had the same issue in the ‘questions.txt’ file. If you choose to proceed with the trials, make note of which questions had a problem and write them down in the session notes at the end of the session. The app will record the subject’s data and immediately upload it to the database whenever the subject clicks the ‘Submit’ button during a trial, so there will be some data that can be preserved and analyzed.
• If you consider the data unusable due to the interruption, you may interrupt the session and ask the subject to re-do the problematic story after you’ve troubleshooted it. To do this, follow the following steps. First bring the command line window up and enter the keyboard combination ‘Ctrl + C’. If you’re prompted to confirm, enter ‘Y’ and press the Enter key. Make sure you make any necessary corrections to the ‘questions.txt’ file and navigate to ‘\bin\settings.ini’. Open this file with your text editor of choice (f.e. Notepad, or Notepad++). In line 34, make the following change: next_story_from=local. Then, save the settings file and restart the app as you did before. Ask the subject to select however many stories they had left in the previous session and run them through the same setup as before. When asked ‘Are you a new participant?’, ask the subject to click ‘No’ and enter their ID number.

### Problem 3

The startup.bat script fails before the application is launched due to software incompatibility. This corresponds to session set-up step 2.

### Potential solution

Make sure all of the versions for the software used match the versions in the key resources table. Specifically, make sure that you are using Python 3.10.5. If there is a double installation of python, modify the .bat so that it uses «"C:\Users\username\AppData\Local\Programs\Python\Python310\python.exe" -m ensurepip --upgrade».

### Problem 4

The startup.bat script fails with an “ERR_CONNECTION_REFUSED” message. This corresponds to session set-up step 2.

### Potential solution

This is due to a firewall issue. It can be resolved by navigating to Windows Defender Firewall, clicking “allow an app through firewall”. Then, clicking on “Change settings” in the top right, and “Allow another app” on the bottom left. Add “python.exe” from the correct python version, as mentioned in potential solution to problem 3, then give private and public rights to Python.

### Problem 5

The startup.bat script fails due to not finding an associated heart-rate monitor or eye-movement tracking device. This corresponds to session set-up step 2.

### Potential solution

If you plan to run the app without using a heart-rate monitor or eye-movement tracking device, both of which are entirely optional to running the application, ensure that the following settings in the file ‘/bin/settings.ini’ are marked: (lines 76-77) use_eyetracker=0, (lines 88-89) use_hrtracker=0.

## Resource availability

### Lead contact

Further information and requests for resources should be directed to and will be fulfilled by the lead contact, Dr. Alexander Friedman (afriedman@utep.edu).

### Technical contact

Further information and requests for technical issues should be directed to and will be fulfilled by the technical contact, Lara I. Rakocevic (lrakocevic@miners.utep.edu).

### Materials availability

This study did not generate new unique reagents.

### Data and code availability

All code relating to running this application can be found at HUMANS App GitHub Repository (https://github.com/rjibanezalcala/HUMANS). Please see the associated *Cell Reports Methods* manuscript for access instructions for datasets and additional code generated during this study.

## Acknowledgments

This project was supported by the 10.13039/100000001NSF/CAREER (#2235858), NIH/10.13039/100000026NIDA (#R01DA058653), and the 10.13039/100014037National Defense Science and Engineering Graduate Fellowship (NDSEG) to L.I.R.

## Author contributions

Conceptualization, A.F. and K.A.G.; data curation, L.I.R.; formal analysis, L.I.R.; funding acquisition, A.F., K.A.G., and L.I.R.; investigation, A.F., K.A.G., L.I.R., and R.I.A.; methodology, A.F., K.A.G., L.I.R., and R.I.A.; project administration, A.F.; software, A.F., L.I.R., and R.I.A.; supervision, A.F.; validation, A.F. and L.I.R.; visualization, L.I.R. and R.I.A.; writing – original draft, A.F., L.I.R., and R.I.A.; writing – review and editing, A.F., K.A.G., L.I.R., and R.I.A.

## Declaration of interests

The authors declare no competing interests.
